# The Aggregation and Dissolution of Citrate−Coated AgNPs in High Ammonia Nitrogen Wastewater and Sludge from UASB−Anammox Reactor

**DOI:** 10.3390/ijerph19159502

**Published:** 2022-08-02

**Authors:** Jiachao Jiang, Xin Wang, Yuanyuan Zhang, Jiageng Zhang, Xiujun Gu, Shilong He, Shuo Duan, Jianli Ma, Lizhang Wang, Ping Luo

**Affiliations:** 1 School of Environment Science and Spatial Informatics, China University of Mining and Technology, 1 Daxue Road, Xuzhou 221116, China; wx960910@163.com (X.W.); zyy200513@163.com (Y.Z.); zhangjg1206@163.com (J.Z.); ts21160123p31@cumt.edu.cn (X.G.); hslongrcees@163.com (S.H.); wlzh0731@126.com (L.W.); 2 Jiangsu Key Laboratory of Resources and Environmental Information Engineering, China University of Mining and Technology, Xuzhou 221116, China; 3 Key Laboratory for Deep Processing of Major Grain and Oil of Ministry of Education, College of Food Science and Engineering, Wuhan Polytechnic University, Wuhan 430023, China; duanshuo0718@whpu.edu.cn; 4 Solid Waste and Soil Environment Research Centre, Tianjin Academy of Eco–Environmental Sciences, Tianjin 300191, China; majianguang@163.com

**Keywords:** silver nanoparticles, aggregation, dissolution, sludge, high ammonia nitrogen wastewater

## Abstract

Silver nanoparticles (AgNPs) are released into the sewage pipes and ultimately wastewater treatment plants during manufacturing, use, and end–life disposal. AgNPs in wastewater treatment plants aggregate or dissolve, and may affect the microbial community and subsequent pollutant removal efficiency. This study aims to quantitatively investigate the fate of AgNPs in synthetic high ammonia nitrogen wastewater (SW) and sludge from an up–flow anaerobic sludge blanket (UASB) anammox reactor using a nanoparticle tracking analysis (NTA), dynamic light scattering (DLS), transmission electron microscope (TEM), and atomic absorption spectroscopy (AAS). Results showed that 18.1 mM NH_4_^+^, 2.11 mM Mg^2+^ in SW caused less negative zeta potential (ζ−potential, −18.4 vs. −37.4 mV), aggregation (388.8 vs. 21.5 nm), and settlement (80%) of citrate−coated AgNPs (cit−AgNPs) in 220 min. The presence of 18.5 mM Cl^−^ in SW formed AgCl_2_^−^, AgCl_(aq)_ and eventually promoted the dissolution (9.3%) of cit−AgNPs. Further exposure of SW−diluted AgNPs to sludge (42 mg L^−1^ humic acid) and induced a more negative ζ−potential (−22.2 vs. −18.4 mV) and smaller aggregates (313.4 vs. 388.8 nm) due to the steric and hindrance effect. The promoted Ag dissolution (34.4% vs. 9.3%) was also observed after the addition of sludge and the possible reason may be the production of Ag(NH_3_)_2_^+^ by the coexistence of HA from sludge and NH_4_^+^ from SW. These findings on the fate of AgNPs can be used to explain why AgNPs had limited effects on the sludge−retained bacteria which are responsible for the anammox process.

## 1. Introduction

Silver nanoparticles (AgNPs) have the unique property of conductivity, sensory and antibacterial and therefore have been utilized in diverse domains [1,2,3,4,5]. According to the Nanotechnology Products Database, AgNPs have been used in 1052 different product types, 64 countries, and 15 industries, i.e., medical, textile, electronics, cosmetics, packaging, and coatings [6]. It is inevitable that these AgNPs will be released into the sewage pipes and ultimately wastewater treatment plants during manufacturing, use, and end−life disposal [7,8]. The concentration of AgNPs in sewage and sludge was found as 6~240 ng L^−1^ and 0.0043~14 mg kg^−1^ respectively [9,10,11,12].

Although the environmental behavior of AgNPs has received intensive attention in the last decade, the behavior of nanomaterials in real matrices, especially in wastewater, is generally poorly investigated. This information is needed to be considered when exploring their fate and toxicity, which highly depend on the complex matrices of the aqueous constituents in the environment [13,14]. Wastewater is one of the most complex matrices. This study focused on one type of high ammonia nitrogen content wastewater and sludge from the anammox process. The anammox process is an alternative nitrogen removal process where autotrophic anammox bacteria directly convert ammonium to N_2_, a harmless gas, using nitrite as the electron acceptor under anaerobic condition [15]. It requires less oxygen, less sludge yield, and less or no organic carbon sources than traditional nitrogen removal processes and therefore has been recognized as an indispensable part in both the sidestream and mainstream in the ‘new concept sewage treatment plants’ of the future in China [16].

Recently, research on the impacts of AgNPs on the anammox process were conducted [17,18]. AgNPs resistance studies showed that the gradually increased AgNPs (1, 10, 50 mg L^−1^) showed no effects on anammox activity, reactive oxygen species production, or cell membrane integrity [19]. Thereafter no further adverse effects on the nitrogen removal performance and on the relative abundance of *Cadidatus Kuenenia* inhabiting in granules were observed [16]. Li et al. (2019) showed that 1 mg L^−1^ AgNPs had no effect on total nitrogen removal while 10 mg L^−1^ AgNPs had an inhibiting effect on the nitrogen removal in the unplanted subsurface−flow constructed wetlands [20]. Peng et al. (2019) suggested that the exposure of a high concentration of AgNPs in the anammox process can even improve nitrogen removal [21]. This is contrary to our prediction since the toxicity of AgNPs on bacteria, algae, and mammals is well−acknowledged [22,23]. Studies have demonstrated that AgNPs had inhibition on nitrification [24], enzyme activity, the number of nitrifying bacteria [25], the abundance of the amoA gene, the gross nitrification rate, and ultimately inhibited nitrogen removal [26,27]. It is anticipated that the possible reason of AgNPs resistance on anammox granules is due to AgNPs aggregation. However, to the best of our knowledge, there is no publication covered the detailed fate of AgNPs in an anammox reactor so far.

This research hence aims to investigate how SW and sludge from the anammox process affect the AgNPs aggregation and dissolution, respectively, or in combination. Citrate coating was selected since it is the most used AgNPs (cit−AgNPs), which is stabilized by steric repulsion. Its fate is very sensitive to the matrix components [28]. The changes in cit–AgNPs size, number concentration, and zeta potential (ζ−potential) were monitored before and after exposure to the SW, with or without sludge. The total interaction energy (V_Total_) between particles was then calculated by the Derjaguin–Landau–Verwey–Overbeek (DLVO) theory to explain the aggregation status. The amount of dissolved Ag was measured and its theoretical equilibrium speciation was calculated by visual MINTEQ (version 3.1) modelling. It is believed that these results will fill the knowledge gap of the fate of AgNPs in an up–flow anaerobic sludge blanket (UASB) anammox reactor, which is critical to understand the impact of AgNPs on the anammox process.

## 2. Materials and Methods

### 2.1. Chemicals and Reagents 

All chemicals and reagents, e.g., silver nitrate (AgNO_3_), sodium borohydride (NaBH_4_), and aqueous solution of trisodium citrate (TSC) were of analytical grade. They were purchased from Sinopharm Chemical Reagent Co. Ltd. (Shanghai, China). All glassware used in this study was first acid−washed by soaking in a 30% HNO_3_ solution overnight, then rinsed three times in deionized (DI) water and allowed to dry in an oven (80 °C).

### 2.2. Anaerobic Ammoxidation Sludge and Synthetic High Ammonia Nitrogen Wastewater (SW)

The anaerobic ammoxidation sludge was collected from an UASB–anammox reactor which operated at 35 °C for more than 300 days in the lab. The average granule diameter of inoculated sludge was 2.69 mm, the biomass concentration was 17.6 g volatile suspended solids (VSS) per liter and the ratio of VSS/suspended solids (SS) was 0.28. The SW used to preserve the sludge employed N–NH_4_^+^ and N–NO_2_^−^ (1.32:1) in the forms of (NH_4_)_2_SO_4_ and NaNO_2_ as substrates. Other compositions of the SW were nutrient and trace elements. The nutrient components included 0.25 g L^−1^ KHCO_3_, 0.01 g L^−1^ KH_2_PO_4_, 0.3 g L^−1^ MgSO_4_·7H_2_O, and 0.0056 g L^−1^ CaCl_2_·2H_2_O. Trace elements contained 15 g L^−1^ EDTA, 5 g L^−1^ FeSO_4_, 0.43 g L^−1^ ZnSO_4_·7H_2_O, 0.24 g L^−1^ CoCl_2_·6H_2_O, 0.99 g L^−1^ MnCl_2_·4H_2_O, 0.25 g L^−1^ CuSO_4_·5H_2_O, 0.22 g L^−1^ NaMoO_4_·2H_2_O, and 0.19 g L^−1^ NiCl_2_·2H_2_O [29]. The dissolved oxygen of SW was 0.22 mg L^−1^. The pH of SW without and with sludge was 7.7 and 8.7, respectively. The initial ionic strength (IS) of SW was 100 mM.

### 2.3. Synthesis and Characterization of AgNPs

Electrostatically stabilized cit–AgNPs were synthesized following the method reported by Shekhar et al. (2014) with minor modifications [30]. The protocol relied on the reduction in AgNO_3_ using NaBH_4_ as a primary reductant and TSC, which has a dual role to act as a reducing agent as well as stabilizing agent [30]. The reduction processes were started by mixing 8 mM NaBH_4_ and 3.2 mM TSC for 30 min (60 °C) in the dark with continuous and vigorous stirring to ensure a homogenous solution. Then 20 mM AgNO_3_ was added drop–wisely while the temperature was further raised to 90 °C. The pH was adjusted to 10.5 using 0.1 M NaOH until the color changed to bright yellow. Finally, the synthesized AgNPs were purified by centrifugation (8500 r min^−1^, 15 min), diluted in distilled water, and stored in the dark at 4 °C. 

The UV–visible absorption spectra of synthesized AgNPs were acquired using an ultraviolet–visible spectrophotometer (UV–vis, SP–756P, Shanghai Spectrum, Shanghai, China) in the energy range of 200–600 nm. The dry size of AgNPs were determined using a transmission electron microscope (TEM, Tecnai G2 F20, PEI, Hillsboro, OR, USA) at 200 kV. Eight representative images of AgNPs (111 nanoparticles in total) were analyzed by the NanoMeasurer software (version 1.2). The hydrodynamic particle sizes of AgNPs were measured by nanoparticle tracking analysis (NTA, Malvern, UK) following dispersion in DI water. The ζ–potential was recorded using dynamic light scattering (DLS, zetasizer nano ZS90, Malvern, UK). The mass concentration was determined by atomic absorption spectroscopy (AAS, A3AFG, Persee, Beijing, China) after acid digestion. Further information with detailed methods were described in Luo et al. [31].

### 2.4. Experiments Design

To study the effect of SW on the aggregation and dissolution of AgNPs, sonicated stock AgNPs were dispersed into SW in serial dilution factors, i.e., 0, 10, 20, 50, 100, and 500 and fixed on an orbital shaker (140 rpm, 35 ± 0.5 °C). After being shaken for 0, 30, 60, 90, 120, 150, 180, and 210 min, 30 mL samples were taken for the following analysis; 10 mL samples were taken for size and number concentration analysis using NTA, and 10 mL samples were taken for spectra analysis using UV–vis. The last 10 mL samples were centrifuged (8000× *g* rpm, 20 min) to isolate dissolved Ag, fixed in 5% nitric acid for Ag mass concentration analysis using AAS. The effect of SW on the ζ–potential changes over time (0, 10, 20, 30, 40, and 50 min) were performed by dispersing the sonicated stock AgNPs in SW at the optimum dilution factor and then analyzed by DLS. Control experiments with DI water were also carried out for comparison.

To further study the effect of anaerobic ammoxidation sludge from the anammox process on the aggregation and dissolution of AgNPs, sonicated stock AgNPs were firstly diluted in SW at the optimum dilution factor and mixed with sludge (0.02 g mL^−1^). After being shaken for 0, 30, 60, 90, 120, 150, 180, and 210 min (140 rpm, 35 ± 0.5 °C), 30 mL samples were taken for NTA, UV–vis, and AAS analysis. NTA and UV–vis samples (10 mL each) were centrifuged (5000× *g* rpm, 10 min) to preclude the sludge particles. AAS samples (10 mL) were firstly centrifuged (8000× *g* rpm, 20 min) to isolate dissolved Ag and further fixed with 5% nitric acid. The effect of sludge on the ζ–potential changes over time (0, 10, 20, 30, 40, and 50 min) were performed by dispersing sonicated stock AgNPs in SW and sludge (0.02 g mL^−1^), and shaking the mixture in an orbital shaker (140 rpm, 35 ± 0.5 °C) for 5 min; then they were analyzed by DLS. Similar experiments with DI water and sludge were also undertaken for comparison.

### 2.5. Size Aggregation Rate Calculation

Size measurements were performed after mixing AgNPs with SW with or without sludge for 0, 30, 60, 90, 120, 150, 180, and 210 min respectively. Each sample was continuously measured for three times by NTA and each measurement lasted for 30 s. The size aggregation rate was calculated by the hydrodynamic size measured at different timing points following the method reported by Wang et al. (2019) with slight modifications [32]. The aggregation rate (*k*) was expressed as the slope ΔD/Δt (nm min^−1^), where ΔD is the increase in diameter (nm) of AgNPs and Δt is the time range (min) between measurements.

### 2.6. Theoretical Equilibrium Speciation Calculation

The software visual MINTEQ (version 3.1) modelling was used to calculate the theoretical equilibrium speciation of SW (Table 1) dissolved Ag in SW, with or without sludge. Given the complex matrices of sludge, the calculation of the chemical speciation of dissolved Ag with full components of sludge was rather difficult since the bacteria and extracellular polymeric substance were not in the software visual MINTEQ. HA only was selected to represent the effect of sludge.

### 2.7. DLVO Analysis

DLVO was used to estimate the stability of AgNPs by calculating the total interaction energy (*V_Total_*) between particles, including van der Waals attraction (*V_VDW_*) and double–layer repulsion forces (*V_EDL_*) [33]. The *V_Total_* was calculated by the Equations (1)–(4) based on the following assumptions: the surface potential was estimated by *ζ–potential; a_p_*_1_ *= a_p_*_2_, *ζ_p_*_1_ *= ζ_p_*_2_ since the suspension comprises AgNPs only.

*V_Total =_ V_VDW_ + V_EDL_* (1)

(2) $$V_{VDW}=-\frac{A_{131}a_{p1}a_{p2}}{6D^{2}\left(a_{p1}+a_{p2}\right)}{\left[1-\frac{5.32D}{\lambda }\ln\left(1+\frac{\lambda }{5.32}\right)\right]}^{-1}\;$$

(3) $$V_{EDL}=\pi \varepsilon_{r}\varepsilon_{0}\frac{a_{p1}a_{p2}}{\left(a_{p1}+a_{p2}\right)}\left\{2\zeta_{p1}\zeta_{p2}ln\left[\frac{1+\exp\left(-\kappa D\right)}{1-\exp\left(-\kappa D\right)}\right]+\left(\zeta_{p1}{}^{2}+\zeta_{p2}{}^{2}\right)ln\left[1-exp\left(-2\kappa D\right)\right]\right\}\;$$

(4) $$\kappa^{-1}=\sqrt{\frac{\varepsilon_{r}\varepsilon_{0}k_{B}T}{2N_{A}Ie^{2}}}\;$$

In Equation (2), *A*_131_ is the Hamaker constant of Cit–AgNPs (3.7 × 10^−20^ J), *D* is the particle spacing, *a_p_*_1_ and *a_p_*_2_ are the particle radius, *λ* is the characteristic wavelength of interaction (10^−7^ m) [34]. In Equation (3), *ε_r_* is the relative dielectric constant of water (78.5 C^2^·N^−1^·m^−2^), *ε*_0_ is the dielectric constant of vacuum (8.85 × 10^−12^ C^2^·N^−1^·m^−2^), *ζ_p_*_1_ and *ζ_p_*_2_ are the *ζ–potential* of particles, *κ* refers to the Debye–Huckel parameter [35]. In Equation (4), *k_B_* is the Boltzmann constant (1.38 × 10^−23^ J/K), *T*(K) is the absolute temperature. *κ* is a function of the electron charge (*e*), the IS (*I*, mol·L^−1^), and Avogadro’s constant *N_A_* (6.02 × 10^23^ mol·L^−1^) [36].

### 2.8. Statistical Analysis

The experiments of each treatment and blank control were carried out in triplicate. Analysis of variance (ANOVA) and least significant difference (LSD) test were performed using the origin software (2018b 9.55) to analyze the statistical differences between treatments at the significance level of *p* < 0.05. Data are expressed as arithmetic means and standard deviations.

## 3. Results

### 3.1. AgNPs Characterization

**Figure 1:**
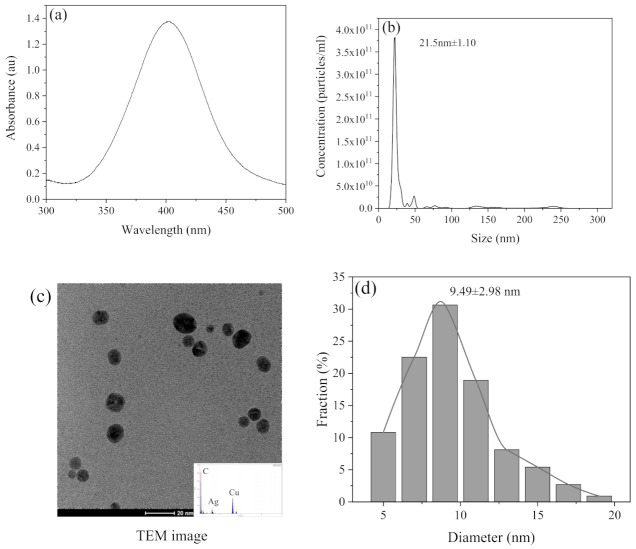
The physico–chemical properties of synthesized cit–AgNPs, such as absorption spectra measured by UV–vis (**a**), hydrodynamic size distribution measured by NTA (**b**), TEM image with EDX (**c**), and dry size obtained by TEM image analysis (**d**).

The physicochemical properties of cit–AgNPs are shown in Figure 1. The strong surface plasmon resonance (SPR) band with a peak of 405 nm suggested Ag nanoparticles were synthesized. Single peak from NTA size distribution (Figure 1b) indicated that mono–dispersed AgNPs were successfully synthesized. The mean hydrodynamic size of AgNPs measured by NTA was 21.5 ± 1.10 nm while the dry particle size measured by TEM image analysis was 9.49 ± 2.98 nm (Figure 1c,d). The ζ–potential measured by DLS was −37.4 mV. The mass stock concentration measured by AAS was 84.0 mg L^−1^ and the number concentration measured by NTA was 8.50 × 10^11^ particles mL^−1^**.**

### 3.2. Changes in ζ–Potential after Exposure to SW and Sludge

**Figure 2:**
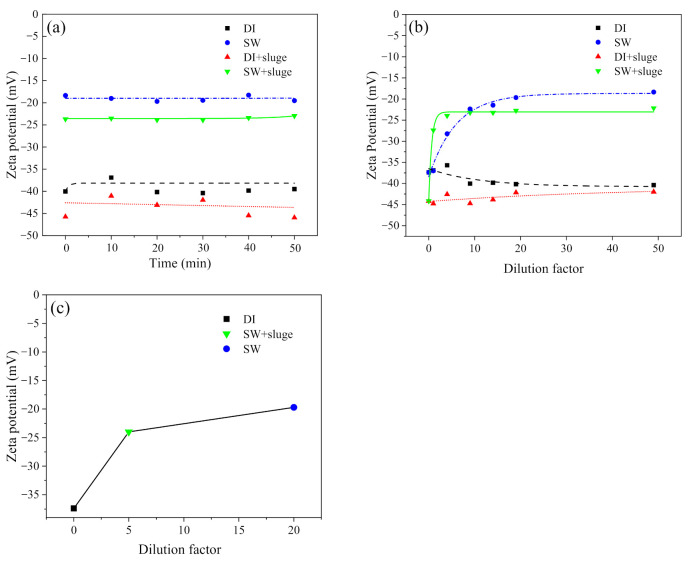
The effect of exposure time (**a**) and dilution at 220 min (**b**) on the ζ–potential changes after exposing cit–AgNPs (50× dilution, 1.7 × 10^10^ particles ml^−1^, 1.68 mg L^−1^) in DI and SW, with or without sludge, respectively. The relationship between plateaued ζ–potential and the minimum dilution required to reach the plateau were shown in (**c**). Notes: pH and IS in SW was 8.7 and 100 mM, respectively. HA in sludge was 42 mg L^−1^.

Figure 2a shows the effect of exposure time on the ξ–potential changes after exposing AgNPs in DI and SW, with or without sludge. The ζ–potential changes were completed within 5 min, maybe less, since the preparation time for DLS measurement was about 5 min. Then they were constantly stable through the experiment time (50 min). Dilution in DI water showed no change in ζ–potential, remaining at around −37.4 mV. The dilution in SW induced less negative ζ–potential (−18.4 mV), being close to the ζ–potential of SW (−16.7 mV). Further addition of 0.02 g L^−1^ sludge led to more negative ζ–potential for both SW–diluted (−18.4 to −22.2 mV) and DI–diluted AgNPs (−37.4 to −42.0 mV) (Figure 2a).

The effect of dilution on the ζ–potential changes after 50 min exposure is shown in Figure 2b. The ζ–potential of AgNPs exposing to DI water, with or without sludge, fluctuated a bit at low dilution (<10×) but was generally stable along the increased dilution factor. The ζ–potential of AgNPs exposing to SW (4.25 × 10^10^ particles mL^−1^, 4.20 mg L^−1^) generally increased along the increased dilution and plateaued with 20× dilution. However, addition of sludge in SW–diluted AgNPs achieved the plateau at 5× dilution (1.7 × 10^11^ particles mL^−1^, 16.8 mg L^−1^). Figure 2c presents the relationship between plateaued ζ–potential and the minimum dilution required to reach the plateau. Cit–AgNPs stabilized with a more negative ζ–potential were plateauing at a higher maximum concentration.

### 3.3. Changes in AgNPs Size after Exposure to SW and Sludge

**Figure 3:**
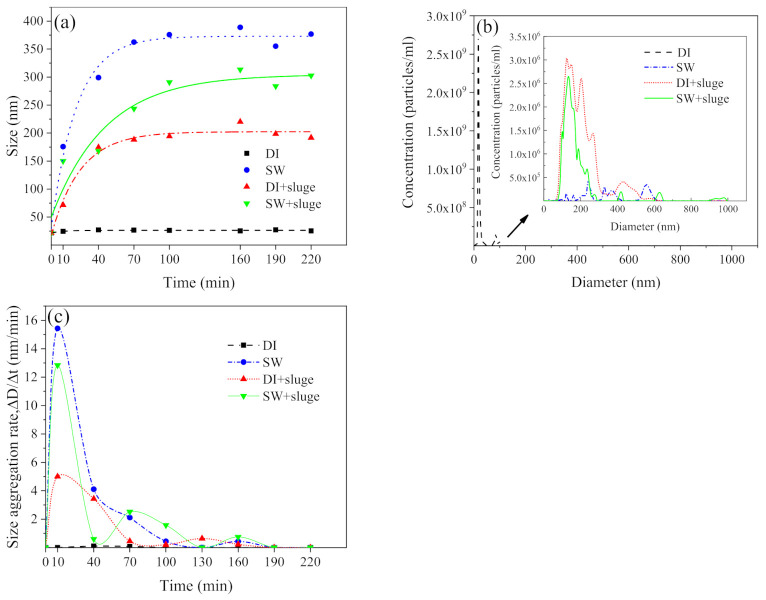
The effect of exposure time on the hydrodynamic size (**a**), size distribution (**b**), and aggregation rate (**c**) of DI− and SW−diluted cit−AgNPs (50× dilution, 1.7 × 10^10^ particles ml^−1^, 1.68 mg L^−1^), with or without sludge. Notes: pH and IS in SW was 8.7 and 100 mM, respectively. HA in sludge was 42 mg L^−1^.

**Figure 4:**
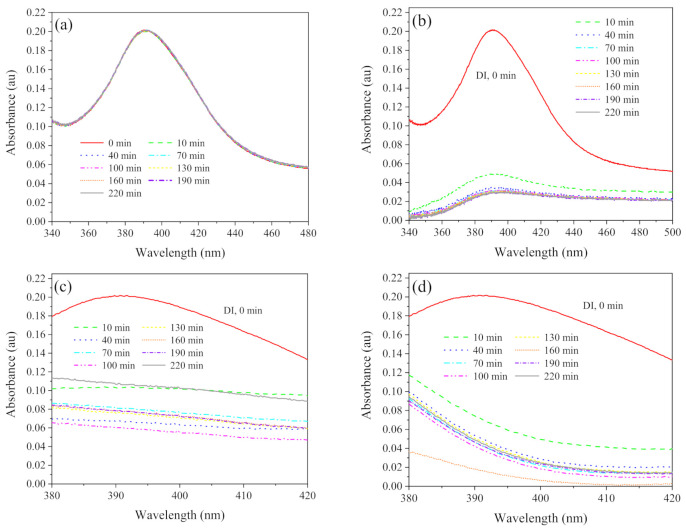
The effect of exposure time on the UV−vis spectra changes after exposing cit−AgNPs (50× dilution, 1.7 × 10^10^ particles ml^−1^, 1.68 mg L^−1^) in DI (**a**), SW (**b**), DI with sludge (**c**), and SW with sludge (**d**), respectively. Notes: pH and IS in SW was 8.7 and 100 mM, respectively. HA in sludge was 42 mg L^−1^.

Figure 3 and Figure 4 show the effects of exposure time on the aggregation of SW−diluted AgNPs (50× dilution, 1.7 × 10^10^ particles mL^−1^, 1.68 mg L^−1^), with and without sludge. AgNPs exposed in DI water (control) showed a stable size (21.5 nm) over the experimental period (Figure 3a). However, AgNPs exposed to the SW at the same concentration were observed with the hydrodynamic size of 176 nm, further to 299 nm after 40 min, and eventually stabilized at around 388.8 nm after 50 min (Figure 3a). Figure 3b shows that SW−diluted AgNPs had multiple peaks between 240 nm and 555 nm while DI−diluted AgNPs remained as a single peak of 18 nm. Furthermore, the presence of sludge induced significant aggregation of DI−diluted AgNPs (220.1 vs. 21.5 nm) whilst it induced smaller aggregates of SW−diluted AgNPs (313.4 vs. 388.8 nm). UV−vis spectra (Figure 4) changes also confirmed the aggregation. Single peak of absorption spectra were observed at 390 nm after AgNPs exposed to DI water for over 220 min, while widened and reduced spectra were observed for AgNPs exposed in SW, SW with sludge, and DI with sludge.

Figure 3c shows the calculated aggregation rate (ΔD/Δt, nm min^−1^) of AgNPs. It reached the maximum (15.43 nm min^−1^) after exposure to SW for about 40 min, then significantly decreased in the next 70 min, and eventually stabilized at 0.44 nm min^−1^ within 100 min. On the contrary, the aggregation rate of DI−diluted AgNPs was relatively low (0.14 nm min^−1^). After the addition of sludge, the maximum aggregation rate (Figure 3c) of DI−diluted AgNPs increased to 5.0 nm min^−1^ while that of SW−diluted AgNPs reduced to 12.8 nm min^−1^.

**Figure 5:**
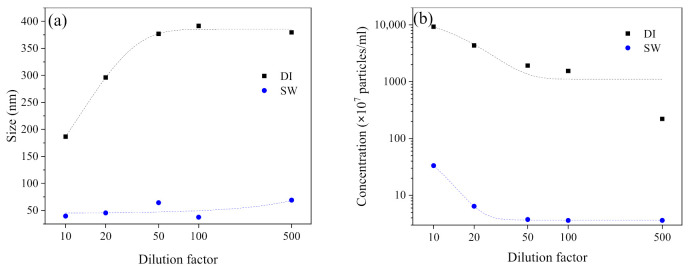
The effect of dilution on the size (**a**) and concentration (**b**) changes after exposing cit−AgNPs in DI and SW (pH = 8.7, IS = 100 mM) for 220 min.

Further to the effect of exposure time, the effect of dilution on the AgNPs aggregation profile was also studied (Figure 5). The experiment period of 220 min was selected since our previous results showed that the aggregation stabilized within 220 min. The increased dilution with SW led to the corresponding increased size of AgNPs (Figure 5a). Eventually the size stabilized at around 388.8 nm at 50× dilution (IS = 100 mM, 1.7 × 10^10^ particles mL^−1^, 1.68 mg L^−1^). While the size remained at around 21.5 nm for DI−diluted AgNPs through the dilution process. Referring to the concentration changes, both SW− and DI−diluted AgNPs showed decreased concentration along with the increased dilution factor (Figure 5b). DI−diluted AgNPs decreased linearly while the SW−diluted AgNPs decreased exponentially. The concentration of SW−diluted AgNPs in SW were constantly below DI−diluted AgNPs. Therefore, the change in AgNPs in SW was attributed to not just the dilution but also to the aggregation and possible sedimentation (Figure 5b). The flattened trend for SW−diluted ones between 100× and 500× dilution factor was also noted (Figure 5b). The possible reason is that AgNPs concentration (10^7^–10^8^ particles mL^−1^) reached the detection limit of NTA, i.e., approximately 10 particles on the screen [37].

### 3.4. DLVO Analysis of AgNPs

**Figure 6:**
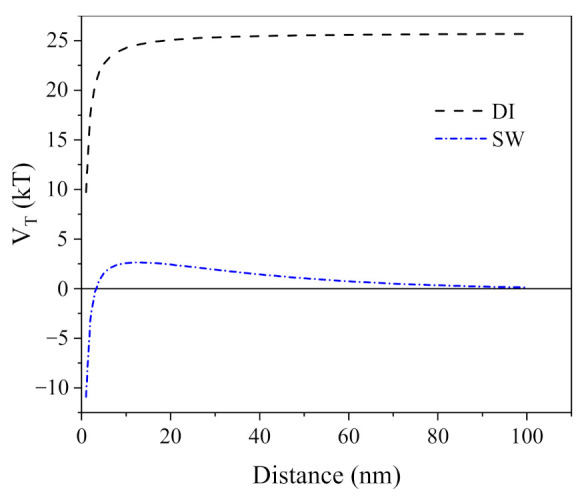
The DLVO interaction energy spectrum of DI and SW (pH = 8.7, IS = 100 mM) diluted cit−AgNPs.

The DLVO interaction energy spectrum of DI− and SW−diluted AgNPs is shown in Figure 6. The energy spectrum of AgNPs in the presence of sludge were not shown since DLVO theory uses ζ−potential to explain the forces between charged surfaces through a liquid medium. The sludge is not applicable for the DLVO theory [38] since it contains lots of solid particles and HA, which induced the bridging effect. In between the particle spacing of 0–100 nm, the total potential repulsion energy between DI−diluted AgNPs was constantly positive (>22 K_B_T) since the electrostatic repulsion force (1.063 × 10^−19^ to 1.062 × 10^−19^ J) was always higher than the van der Waals force (−6.6 × 10^−21^ to −6.6 × 10^−25^ J) (Figure 6). For SW−diluted AgNPs, the total potential repulsion energy dramatically dropped to one tenth (2.65 K_B_T) since the increased IS (3.98 × 10^−5^ to 100 mM) induced less negative ζ−potential (Figure 2a). This can be used to explain the aggregation profiles observed with NTA (Figure 3a and Figure 5), where AgNPs in SW (IS = 100 mM) tend to cross the energy barrier, then collide and aggregate together (Figure 3a).

### 3.5. Theoretical Equilibrium Speciation of Dissolved AgNPs

**Figure 7:**
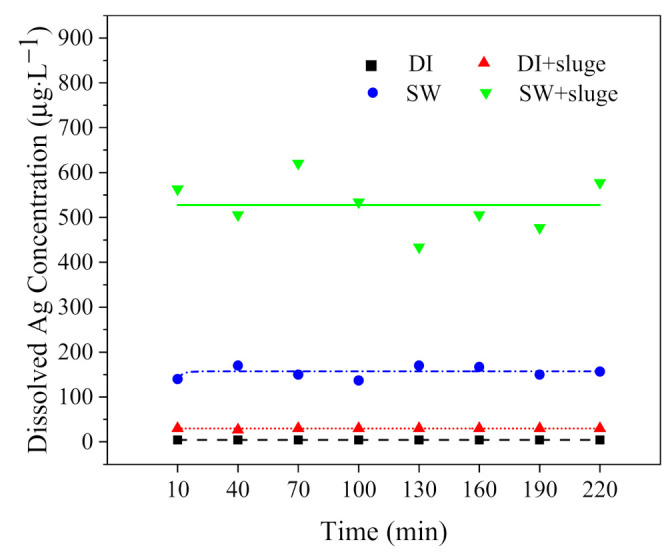
The effect of exposure time on the dissolved Ag after exposing cit−AgNPs (50× dilution, 1.7 × 10^10^ particles ml^−1^, 1.68 mg L^−1^) in DI and SW, with or without sludge, respectively. Notes: pH and IS in SW was 8.7 and 100 mM, respectively. HA in sludge was 42 mg L^−1^.

**Figure 8:**
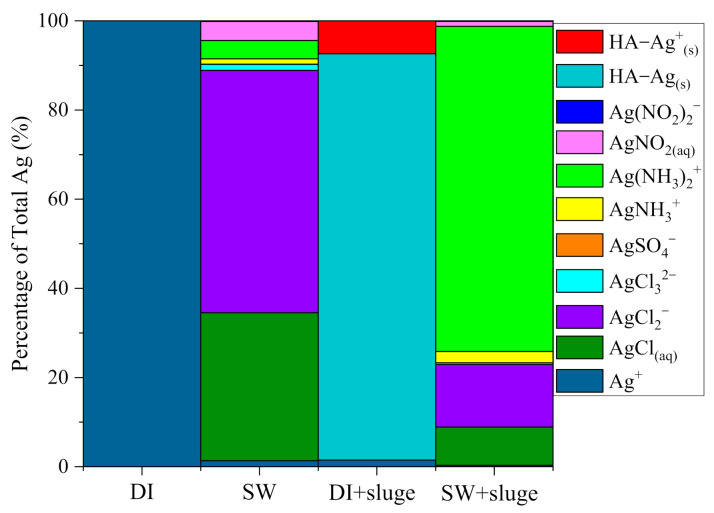
The calculated theoretical equilibrium speciation for dissolved Ag after exposing cit−AgNPs (50× dilution, 1.7 × 10^10^ particles mL^−1^, 1.68 mg L^−1^) in DI and SW, with or without sludge, respectively, using visual MINTEQ, version 3.1. Notes: pH and IS in SW was 8.7 and 100 mM respectively. HA in sludge was 42 mg L^−1^.

The concentrations of released ionic Ag over 220 min are shown in Figure 7. The dissolved Ag was stable over the experiment time. The dissolution percentage of AgNPs in DI, SW, and DI with sludge, and SW with sludge was 0.3% (4.3 μg L^−1^), 9.3% (156.7 μg L^−1^), 1.8% (30.0 μg L^−1^), and 34.4% (577.5 μg L^−1^), respectively. The ranking is as follows: DI < DI with sludge < SW < SW with sludge. The results indicate that the addition of SW and sludge both significantly promoted the dissolution of AgNPs. The calculated theoretical equilibrium speciation for dissolved Ag in different medium are shown in Figure 8.

**Figure 9:**
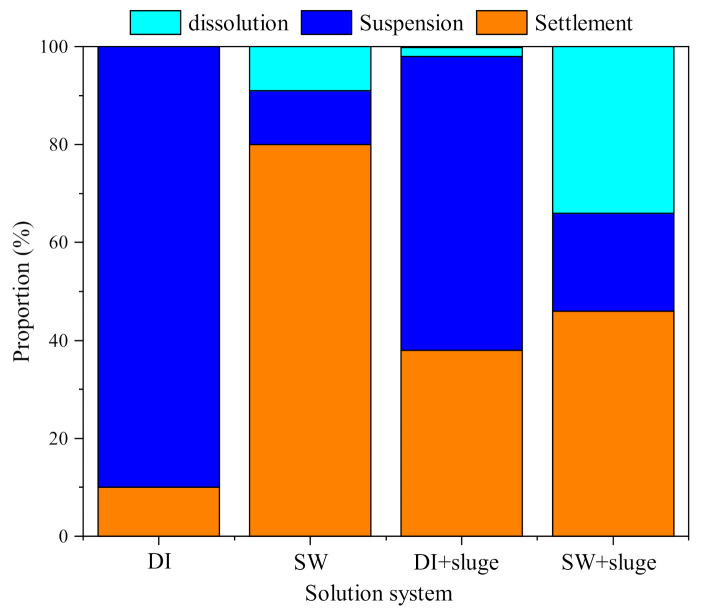
The mass balance of DI and SW−diluted cit−AgNPs (50× dilution, 1.7 × 10^10^ particles ml^−1^, 1.68 mg L^−1^) with and without sludge after 220 min, such as proportions of settlement, dissolution, and suspension. Notes: pH and IS in SW was 8.7 and 100 mM, respectively. HA in sludge was 42 mg L^−1^.

Figure 9 shows the mass balance of DI and SW−diluted AgNPs with and without sludge after 220 min, such as proportions of suspension, dissolution, and settlement. DI−diluted AgNPs were mainly suspended in the supernatant (90%). About 9.7% settled or absorbed on the container, while 0.3% Ag dissolved as free Ag^+^. The exposure of AgNPs (21.5 nm) to SW caused aggregation (388.8 nm) and settlement (80%). A total of 9.3% of Ag dissolved as ionic Ag, such as AgCl_2_^−^ (54.3%) and AgCl_(aq)_ (33.2%). Only 10.7% AgNPs remained in suspension as NTA measurable ones. Further exposure of SW−diluted AgNPs to sludge induced smaller aggregate size (313.4 nm) and less settlement (46%) in comparison with SW−diluted AgNPs (388.8 nm, 80%). AgNPs (34.4%) dissolved mainly as Ag(NH_3_)_2_^+^ (72.9%), AgCl_2_^−^ (14.0%), and AgCl_(aq)_ (8.6%). Around 20% of AgNPs retained in suspension after 100 min. However, exposure of DI−diluted AgNPs to sludge led to significant aggregation (220.1 nm) and increased settlement (38%) compared with that in the absence of sludge (21.5 nm, 9.7%). The addition of sludge shifted the free Ag^+^ in DI−diluted AgNPs to HA−Ag_(s)_ (91.1%) and HA−Ag^+^_(s)_ (7.4%) due to the presence of 42 mg L^−1^ HA. Regarding to the timing, the ζ−potential changes, aggregation, and settlement took place within 5, 50, and 100 min, respectively.

## 4. Discussion

### 4.1. Effect of SW on the Aggregation Behavior of AgNPs

The aggregation profiles of cit−AgNPs in SW (Figure 3c) are typical electrostatically stabilized suspension that follows a DLVO−type aggregation with two regimes, i.e., reaction−limited aggregation and diffusion−limited aggregation [39]. It has been well−studied that the cations (NH_4_^+^, Na^+^, K^+^, Mg^2+^, Ca^2+^) from SW (Table 1) played very important roles in cit−AgNPs aggregation. They may form complexation reaction with the carboxyl groups on the surface of the cit−AgNPs, causing surface charge screening, reducing the thickness of the diffuse double layer surrounding around the electrostatically stabilized cit−AgNPs [40]. Regarding NH_4_^+^, Cervantes−Aviles et al. (2019) reported that 90 mg L^−1^ (4.76 mM) NH_4_^+^ slightly increased the mean aggregate size from 40 nm to 75 nm [41]. However, the mechanisms of NH_4_^+^−induced aggregation has not been well−studied.

Many studies have been conducted to compare the cations efficiency on cit−AgNPs aggregation or their critical coagulation concentration (CCC) values. Divalent cations were reported more efficient than monovalent cations [39,42,43]. In between divalent cations, Huynh, and Chen (2011) reported that Ca^2+^ was more efficient since it has a higher propensity to form complexes with citrate molecules comparing with Mg^2+^ [43]. Baalousha et al. (2013) concluded that the mixture contribution of monovalent and divalent cations to the aggregation behavior is additive, α_mixture_ = α_Na + (50 to 65)Ca_ [39]. Feng (2019) reported that the rank was Ca^2+^ > NH_4_^+^ > K^+^ > Na^+^ in the presence of 10 mM Cl^−^ [17]. The literature review suggested that the rank of aggregations efficiency in our study is Ca^2+^ (CCC = 1.6–5 mM) > Mg^2+^ (2.7 mM) > NH_4_^+^ > K^+^ > Na^+^ (48–150 mM) [41,43,44,45,46,47]. The MINTEQ simulation showed that the concentration of counter ions in our SW are 2.1 mM Mg^2+^, 18.1 mM NH_4_^+^, 12.4 mM K^+^, and 22.8 mM Na^+^. Therefore, Mg^2+^ and NH_4_^+^ are possibly the main reasons for aggregation since their concentrations were close to the reported CCC.

Referring to the effect of anions on the cit−AgNPs aggregation, the MINTEQ simulation showed that the anion concentrations in our SW were 23.2 mM NO_2_^−^, 18.5 mM Cl^−^, 2.0 mM SO_4_^2−^, and 0.05 mM CO_3_^2−^. Cl^−^ was reported much more efficient in aggregating cit−AgNPs than others, Cl^−^ > NO_3_^−^ = PO_4_^3−^ > SO_4_^2−^ [39,48]. At a low Cl/Ag ratio, Cl^−^ reacted with Ag^+^ and deposited a solid AgCl layer on the surface of AgNPs, which induces aggregation by its inter−particle bridging effect [28,49,50]. The NO_2_^−^ was proven to be able to induce aggregation through the selective diazo−coupling strategy [51]. The carbonate and sulfate have a high affinity to silver (8.46 × 10^−12^ M^2^, 1.2 × 10^−5^ M^2^) and form layers of Ag_2_CO_3_ and Ag_2_SO_4_, respectively [52], resulting in less negative ζ−potential and aggregation. Afshinnia et al. (2017) reported that the coexistence of counter ions and carbonate anions in SW may lead to a more efficient aggregation, the first step is adsorption of carbonate anions on the surface of cit−AgNPs and the second step is physical surface charge screening by counter ions [53].

### 4.2. Effect of Sludge on the Aggregation Behavior of AgNPs

After being further exposed to 0.02 g L^−1^ sludge, diluted cit−AgNPs were bound to the negatively charged polyanionic HA (42 mg L^−1^) via two different mechanisms. One way is the specific binding of HA to reactive sites on the particles by partial substitution of the small citrate anions. The other way is governed by the properties of the double layer [54,55]. AgNPs subsequently became more negative in both DI water (−42 vs. −37.4 mV) and SW (−22.2 vs. −18.4 mV)−diluted systems in the presence of sludge.

**Figure 10:**
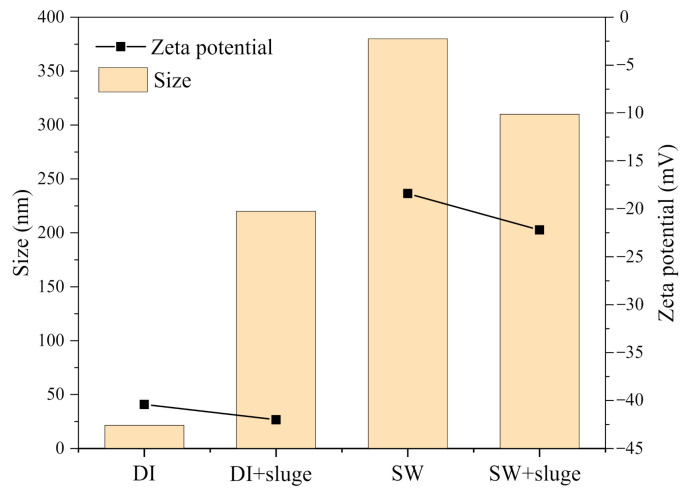
The effect of sludge addition on the size and ζ−potential changes in DI− and SW−diluted cit−AgNPs (50× dilution, 1.7 × 10^10^ particles mL^−1^, 1.68 mg L^−1^). Note: HA in sludge was 42 mg L^−1^.

In the SW sludge AgNPs system, although counter ions in SW (NH_4_^+^, Ca^2+^, Mg^2+^, Na^+^) can neutralize the negative HA [56], the adsorbed HA remaining played a dominant role and led to a more negative ζ−potential (−22.2 vs. −18.4 mV) as well as an increased electrostatic repulsion force between nanoparticles. Meanwhile, the absorbed long molecular HA limited the interaction between cit−AgNPs and SW and caused a steric hindrance effect. The carboxyl and phenolic functional groups in HA can prevent the coagulation and precipitation of cit−AgNPs due to a high complexation ability with metals [57]. Along with the more negative ζ−potential, smaller aggregates (313.4 vs. 388.8 nm) and more suspension (20% vs. 10.7%) after further exposing SW−diluted AgNPs in sludge were thus observed (Figure 10). Nevertheless, the presence of sludge did not fully prevent but only reduce the level of aggregation in SW. This is because (1) according to the DLVO theory, the reduction in the diffuse double layer thickness by cation ions played a major role in the presence of sludge; (2) and the counter ions in SW (IS = 100 mM) unavoidably react with HA, leading to complexations, neutralization, and conformation (coiling).

However, contradictory results were observed in DI sludge AgNPs system (Figure 10), where the ζ−potential of AgNPs became more negative (−42 vs. −37.4 mV) but larger aggregates (220.1 vs. 21.5 nm) were obtained. Less AgNPs (60% vs. 90%) ultimately remained in suspension after 220 min. One of the reasons is the adsorbed HA has a long molecule, which can bridge AgNPs together, eventually aggregate and settle with microbial extracellular polymeric substances [58]. Furthermore, the solid particles in sludge can also lead to heterogeneous aggregations because Brownian motion (perikinetic aggregation), fluid motion (orthokinetic aggregation), and differential settling lead to particle collision between suspended particles and AgNPs [59]. The above effect may successfully overtake the static and steric hindrance effect and resulted aggregation.

### 4.3. Effect of SW and Sludge on the Dissolution Behavior of AgNPs

Exposure of AgNPs in SW dramatically increased the dissolution rate compared with that in DI water (9.3% in SW vs. 0.3% in DI water). This result is consistent with many other studies [41,60]. The possible reason is because the SW has a high IS [49]. Pabel Cervantes−Avilés (2019) found that the increased NH_4_^+^ (2.0 to 5.0 mM) significantly increased the dissolution rate from 26.3% to 48.5% [41]. They also found the increased Cl^−^ (2.0 to 299 mM) increased the dissolution rate from 38.4% to 74.9% by forming AgCl_(s)_ and soluble AgCl_x_^(x − 1)^ [28,49]. Therefore, our SW with similar concentrations of NH_4_^+^ (18.7 mM) and Cl^−^ (18.8 mM) may also result in more dissolution than in DI water. The high content of Cl^−^ (Cl/Ag = 577) in SW induced the formation of AgCl_x_^(x − 1)^, i.e., AgCl_2_^−^ (54.3%) and AgCl_(aq)_ (33.20%), which in turn can accelerate the dissolution of cit−AgNPs [61].

The addition of sludge in DI−diluted AgNPs suggested that the presence of HA (42 mg L^−1^) complexed with DI−diluted AgNPs and formed a coating on the surface of AgNPs, e.g., HA−Ag_(s)_ (91.1%) and HA−Ag^+^_(s)_ (7.39%), which suppressed the dissolution instead [54]. However, the addition of sludge (42 mg L^−1^ HA) to SW−diluted cit−AgNPs significantly promoted the Ag dissolution (9.3% to 34.4%) although the pH increased from 7.7 to 8.7 due to the addition of alkaline sludge. This is an opposite trend to the Nernst law [54,62,63]. Among the dissolved Ag species, 72% of dissolved Ag were Ag(NH_3_)_2_^+^ (Figure 8). Mumper et al. (2013) explained that this is because the increased amount of NH_3_ formed at the pH 8.7 accelerated the oxidation and dissolution of AgNPs through the increased formation of silver amines with surface bound Ag^+^ [64]. However, it was noted that there was no Ag(NH_3_)_2_^+^ formed in SW−diluted AgNPs, indicating the coexistence of HA, NH_4_^+^, and increased pH played a very important role in the accelerated dissolution. Similar results have been observed in other studies. Qian et al. (2021) showed the enhancement of AgNPs dissolution due to the HA in the complex leaching solution (from the sludge generated from coagulation of wastewater) [65]. Zhao et al. (2021) observed the promoted Ag dissolution in the presence of 50 mg L^−1^ suspended sediment and 0.1 M (or 0.3 M) NaCl solution [66]. It was explained that the enhanced Ag dissolution might be due to the dissolved constituents of suspended sediment particles, including dissolved organic matter and inorganic metal ions [66]. Many other publications observed that HA significantly suppressed the Ag dissolution [41,54,60,63]. The possible reason for this difference is because these studies focused on rather simple diluents, e.g., DI water, 2–6 mM NaCl/NaNO_3_ and synthetic municipal wastewater, whilst we employed a complex medium with the coexistence of high IS (100 mM, 18.1 mM NH_4_^+^, 18.5 mM Cl^−^) and a high content of HA (42 mg L^−1^). 

### 4.4. Environmental Indication on the UASB−Anammox Reactor

Agreeing with many studies [10,67,68], the majority of AgNPs aggregated, settled (46–80%), and then retained in the sludge after exposure to SW with and without sludge in the UASB−anammox reactor for 100 min (Figure 9). The induced AgNPs aggregates (from 21.5 to 313.4 or 388.8 nm) significantly reduced the reactive surface area. This might explain why the impact of AgNPs on the anammox process efficiency were rather minor [19,24], since the reduced surface area may induce less, no adverse effects on bacteria responsible for the anammox process. These results can also be used to optimize the treatment of high ammonia nitrogen wastewater in a UASB−anammox reactor. For such wastewater with high content of AgNPs, it is recommended to remove AgNPs by 100 min settling in advance. The majority of AgNPs (80%) would settle down in this step. Then only 10.7% AgNPs and 9.3% ionic Ag (mainly AgCl_2_^−^ and AgCl_(aq)_) in the upper−layer wastewater leave by overflow and enter subsequent treatment units. Furthermore, to guarantee the best settling effect and least Ag dissolution, it is important to prevent the sludge entrance during the settling process since our study showed that the coexistence of sludge and SW induced 3.5−fold more Ag dissolution and about 2−fold more suspended AgNPs.

## 5. Conclusions

Although the environmental behavior of AgNPs has received intensive attention in the last decade, the fate of AgNPs in a UASB−anammox reactor is not fully understood. In the current study, it suggests that high cation ions in high ammonia nitrogen wastewater, especially 2.11 mM Mg^2+^ and 18.1 mM NH_4_^+^, induced AgNPs aggregation (388.8 vs. 21.5 nm) by reducing the thickness of the diffuse double layer. While 18.5 mM anions Cl^−^ may be the predominant reason for promoted dissolution (9.3%), by forming AgCl_2_^−^ and AgCl_(aq)_ which in turn can accelerate the Ag dissolution. Although the addition of UASB−anammox sludge induced the aggregation of DI−diluted AgNPs (220.2 vs. 21.5 nm) through the bridging effect, it resulted in smaller aggregates of SW−diluted AgNPs (313.4 vs. 388.8 nm) instead of by the steric and hindrance effect caused by HA. The coexistence of 42 mg L^−1^ HA and SW may be the dominant reason for the significant dissolution promotion (34.4%), which formed Ag(NH_3_)_2_^+^, AgCl_2_^−^, and AgCl_(aq)_.

While the specific mechanism of aggregation and dissolution was not determined here, the finding of AgNPs aggregates in SW with or without sludge well−explained why AgNPs had limited effects on the sludge−retained bacteria which is responsible for the anammox process. This study mainly focused on the general aggregation and dissolution of AgNPs in sludge and high ammonia nitrogen wastewater from a UASB−anammox reactor. Further research is required to specify the contribution of each component from wastewater and sludge to form the full picture of AgNPs behavior in a UASB−anammox reactor.

## Figures and Tables

**Table 1:** The theoretical equilibrium speciation of synthetic wastewater (SW, pH = 8.7, IS = 100 mM) calculated by visual MINTEQ (version 3.1). Note: % is the weight percentage of species 1 or 2, * is unstable equilibrium state.

Species	Con, mM	Species 1	%	Species 2	%
NH_4_^+^	18.10	NH_4_^+^	96.85	NH_3(aq)_	2.26
Na^+^	22.84	Na^+^	98.50	NaCl_(aq)_	0.59
K^+^	12.42	K^+^	98.79	KSO_4_^−^	0.60
Ca^2+^	0.01	CaEDTA^2−^	61.41	Ca^2+^	32.47
Mg^2+^	2.10	Mg^2+^	84.11	MgSO_4(aq)_	5.56
Zn^2+^	3.89 × 10^−9^	ZnEDTA^2−^	99.99		
Fe^2+^	1.70 × 10^−6^	FeEDTA^2−^	95.59	FeOHEDTA^3−^	4.40
Co^2+^	1.86 × 10^−9^	CoEDTA^2−^	100.00		
Mn^2+^	2.88 × 10^−6^	MnEDTA^2−^	99.93	Mn^2+^	0.04
Cu^2+^	7.36 × 10^−12^	CuEDTA^2−^	99.98	CuOHEDTA^3−^	0.01
Ni^2+^	1.66 × 10^−11^	NiEDTA^2−^	99.99		
Cl^−^	18.50	Cl^−^	98.53	NaCl_(aq)_	0.73
NO_2_^−^	23.19	NO_2_^−^	99.99		
SO_4_^2−^	2.03	SO_4_^2−^	80.47	NH_4_SO_4_^−^	6.66
CO_3_^2−^	0.05	HCO_3_^−^	94.48	H_2_CO_3_*_(aq)_	3.30
MoO_4_^2−^	7.60 × 10^−4^	MoO_4_^2−^	71.19	MgMoO_4(aq)_	28.75
PO_4_^3−^	2.95 × 10^−6^	HPO_4_^2−^	63.88	H_2_PO_4_^−^	10.37
EDTA^4−^	2.08 × 10^−8^	MgEDTA^2−^	53.43	CaEDTA^2−^	19.39

## Data Availability

Not applicable.

## Abbreviation

AAS	Atomic absorption spectroscopy
ANOVA	Analysis of variance
CCC	Critical coagulation concentration
DI	Deionized
DLS	Dynamic light scattering
DLVO	Derjaguin–Landau–Verwey–Overbeek
IS	Ionic strength
LSD	Least significant difference
NTA	Nanoparticle tracking analysis
SS	Suspended solids
SW	Synthetic high ammonia nitrogen wastewater
TEM	Transmission electron microscope
TSC	Trisodium citrate
UASB	Up−flow anaerobic sludge blanket
UV−vis	Ultraviolet−visible spectrophotometer
VSS	Volatile suspended solids
